# Supplementary material for: Electronic single-molecule identification of carbohydrate isomers by recognition tunnelling
Source: Nat Commun. 2016 Dec 21;7:13868. doi: 10.1038/ncomms13868 (PMC5187581; doi:10.1038/ncomms13868)
Supplement: Supplementary Information — Supplementary figures, supplementary tables, supplementary methods and supplementary references. [file ncomms13868-s1.pdf]

Supplementary Methods

D-monosaccharides, maltose, and cellobiose were purchased from Sigma-Aldrich (purity ≥ 99%), L-iduronic acid from Carbosynth. Peptide (Ala-Ser-Ala-NH) and glycopeptide (Ala-Ser(O-GalNAc)-Ala-NH) were custom-synthesized by CPC Scientific. 4-O-sulfated-chondroitin sulfate disaccharides and 6-O-sulfated chondroitin sulfate disaccharides (D0A4 and D0A6) were synthesized in-house. Water was purified in a Milli-Q system with a resistivity of ~18.2 MΩ.cm and total organic carbon of less than 5 ppb. Sample solutions (1 and 100 µM) were prepared in a sodium phosphate buffer (1 mM, pH 7.4). All the solutions were freshly prepared right before measurements. NMR spectra were recorded at Bruker 400 MHz (^{1}H) or Varian 500 MHz NMR at the Arizona State University NMR facility lab. Chemical shifts are given in parts per million (ppm) on the delta scale (δ) and referred either to tetramethylsilane (^{1}H and ^{13}C NMR δ = 0 ppm) or the residual solvent peak. Mass spectra were recorded on Bruker maXis 4G electrospray ionization quadrupole time-of-flight (ESI-Q-TOF) mass spectrometer in Institute of Biodesign, ASU.

Preparation of D0A4 and D0A6

Disaccharides D0A4 and D0A6 were obtained by enzymatic digestion of native CS-A polysaccharides using chondroitinase ABC. Specifically, CS-A (0.1 g, Sigma Aldrich) was digested using chondroitinase ABC enzyme (1 U, Sigma Aldrich) in a digestion buffer (50 mM Tris pH 8.0, 60 mM sodium Acetate, 12 mL) at 37 °C for 24 hours. Disaccharides were separated from other larger fragments using a 2.5 cm × 175 cm size exclusion chromatography column (Bio-Rad Biogel P10 resin). Fractions containing disaccharides were combined and desalted. D0A4 was then separated from D0A6 using analytical strong anion exchange (SAX) HPLC (Waters Spherisorb SAX analytical column) with a salt gradient of 0 to 1 M NaCl at pH 4.0. The structures of the disaccharides were confirmed using ^{13}C-edited heteronuclear single quantum coherence (HSQC) NMR spectroscopy (Supplementary Table 1). ESI-MS analysis for D0A4: found m/z 482.05 (M + H), calcd for $C_{14}H_{20}NNaO_{14}S$ 481.0502; for D0A6: found m/z 482.06 (M + H), calcd for $C_{14}H_{20}NNaO_{14}S$ 481.0502.

Supplementary Table 1. Chemical shift assignments for D0A4 and D0A6

	D0A4		D0A6	
	^{13}C	^{1}H	^{13}C	^{1}H
GluA.1	102.6	5.29, 5.26	104	5.26, 5.21
GluA.2	71.2	3.83	72.2	3.82
GluA.3	67.2	3.94	69	4.13
GluA.4	109.3	5.96	110.1	5.91
GalNAc.1	93.8, 97.5	5.20, 4.76	94.1, 97.8	5.24, 4.75
GalNAc.2	52.4, 55.9	4.36, 4.05	51.6, 55	4.31, 4.02
GalNAc.3	75.6, 78.3	4.29, 4.15	79.5, 82.3	4.15, 3.96
GalNAc.4	80.1, 78.7	4.67, 4.60	71.0, 70.2	4.25, 4.18
GalNAc.5	73.1, 77.3	4.27, 3.84	71.2, 75.5	4.39, 3.98
GalNAc.6	63.8	3.77, 3.69	70.9	4.22, 4.15

NOESY NMR of α-^{M}Glu and β-^{M}Glu

Stock solutions (300 mM) of β ^{M}Glu and α ^{M}Glu were prepared in DMSO-d_6 at room temperature under inert atmosphere. A volume of 0.75 mL solution from each stock solution was used for an individual NMR experiment. ^{1}H, COSY and NOESY were recorded in a Varian 500 MHz NMR at 25°C. The NOE mixing time was set to 400 ms and a total number of scans were set to 16. Each spectrum was recorded for 3 h at 25°C. Data was plotted in VnmrJ 4.0 and exported to adobe illustrator. In case of the α-anomer, a cross peak between H_1 and H_2 was observed (Supplementary Figure 1, a) as expected. For the β-anomer, two NOE cross peaks were observed between H_1 and H_3 as well as between H_1 and H_5 protons (Supplementary Figure 2, b).

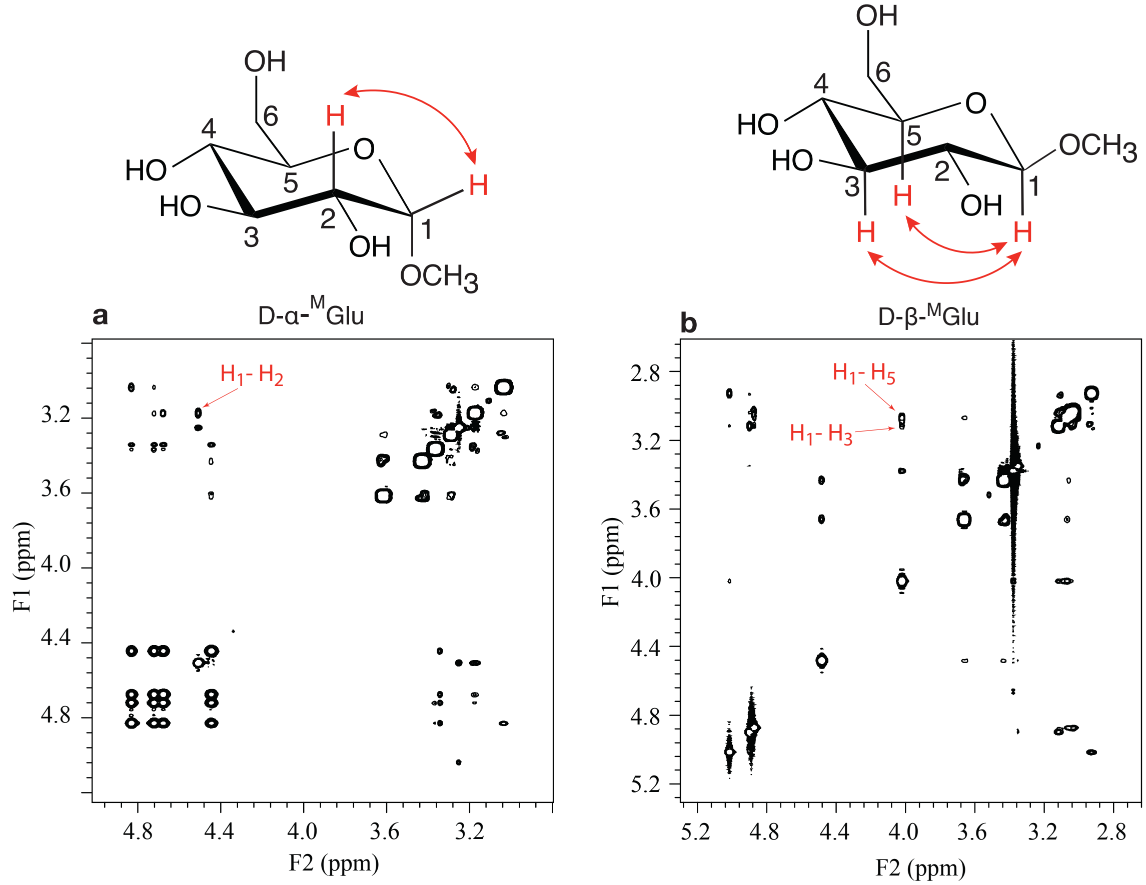

Supplementary Figure 1. ^{1}H–^{1}H NOESY NMR spectra of (a) α-^{M}Glu and (b) β-^{M}Glu

Computation of ICA triplets with α-^{M}Glu and β-^{M}Glu.

DFT calculations were performed using the program Spartan'14 for Windows, Wavefunction Inc. Individual 2D chemical structures of β ^{M}Glu, α ^{M}Glu and ICA were drawn in ChemBioDraw Ultra 14.0, and exported to Spartan'14 to generate the corresponding 3D structures, where hydrogen bonded triplexes were formed by energy minimization using the built-in MMFF94s molecular mechanics. The complex structures were further optimized using B3LYP/6-31G* in water with two sulfurs constrained at a distance of 2.2 nanometer. The final results are shown in Figure 1**d** and **e** (see attached files "Imidazole alfa methylsugar water.pdb" and "Imidazole beta methylsugar water.pdb"for their coordinates in a pdb format).

The hydrogen bonding energy of each triplet was calculated by the total energy of the hydrogen-bonded complex minus the energies of individual components that constitute the complex.

Complexes of ICA with carbohydrates measured by ESI-MS.

By means of ESI-MS, we first obtained characteristic m/z peaks for each individual carbohydrate and ICA in aqueous solution, and then measured the aqueous solutions of ICA mixed with each carbohydrate in 2:1 ratio. Data showed that all monosaccharides and ICA existed as self-associated dimers in solution along with their monomeric form (Supplementary Table 2), ICA formed 1:1 molecular complex ions with all carbohydrates (Supplementary Table 3), and 2:1 molecular complex ions with most of the carbohydrates (Supplementary Table 4). Each molecular ion complex was confirmed by tandem mass spectrometry (MS/MS) and reported as MS/MS product ion in the respective columns.

Experimental details. Solutions of ICA (200 µM) mixed with individual carbohydrates (100 µM) were respectively prepared in water and sparged with argon. Each sample solution was injected into the Bruker maXis 4G electrospray ionization quadrupole time-of-flight (ESI-Q-TOF) mass spectrometer at a 3 µL/min infusion rate via syringe pump. Tandem (MS/MS) mass spectrometry was used to observe product ion peaks from molecular complex ion peaks to confirm the composition of the molecular complex. The ESI source was equipped with a microflow nebulizer needle operated in a positive ion mode. The spray needle was held at ground and the inlet capillary set to -4500 V. The end plate offset was set to -500 V. The nebulizer gas and dry gas (N_2) were set to 1.2 Bar and 1.5 L/min, respectively, and the dry gas was heated to 220°C. In TOF-only mode the quadrupole ion energy was set to 4 eV and the collision energy was set to 1 eV. Collision gas (Ar) was set to a flow rate of 20%. In most cases MS/MS experiments were conducted with a precursor ion isolation width of 3 *m/z* units. However, if other ions were present in this range precursor ion isolation width was set to 1 *m/z* unit. Collision energy was set to 10-20 eV, which was sufficient to fragment non-covalent complexes. Each spectrum was recorded over a time period of 0.5 to 1 min. Typically a spectrum acquired for one minute is an accumulation of 60 separate recorded mass spectra averaged across 1 min time period. Signal to noise ratio greater than three (S/N>3) was used to define the limit of detection. Due to the lack of an acid modifier in the infused solutions, most carbohydrates and molecular complexes were observed as single or multiply sodium ions $[M+nNa-(n-1)H]^+$ rather than as protonated molecular form $[M + H]^+$. Average mass accuracy was within 0.025 Da.

Supplementary Table 2. MS data of individual Carbohydrates and ICA

Analytes	Calculated Monoisotopic Mass	[1]Observed *m/z*	Analytes	Calculated Monoisotopic Mass	[1]Observed *m/z*
D-Galactose ($C_6H_{12}O_6$)	180.0634	203.05, $[M+Na]^+$, (74)<br>225.03, $[M+2Na-H]^+$, (2)<br>383.12, $[2M+Na]^+$, (100)<br>405.10, $[2M+2Na-H]^+$, (2)	D-α-^{M}Glu ($C_7H_{14}O_6$)	194.0790	217.07, $[M+Na]^+$, (67)<br>239.05, $[M+2Na-H]^+$, (2)<br>411.15, $[2M+Na]^+$, (100)<br>433.13, $[2M+2Na-H]^+$, (1)
D-Glucose ($C_6H_{12}O_6$)	180.0634	203.05, $[M+Na]^+$, (87)<br>225.03, $[M+2Na-H]^+$, (2)<br>383.12, $[2M+Na]^+$, (100)<br>405.10, $[2M+2Na-H]^+$, (3)	D-β-^{M}Glu ($C_7H_{14}O_6$)	194.0790	217.07, $[M+Na]^+$, (68)<br>239.05, $[M+2Na-H]^+$, (2)<br>411.15, $[2M+Na]^+$, (100)<br>433.13, $[2M+2Na-H]^+$, (1)
D-Galactosamine ($C_6H_{13}NO_5$)	179.0794	180.09, $[M+H]^+$, (100)<br>202.07, $[M+Na]^+$, (16)<br>359.17, $[2M+H]^+$, (64)<br>381.15, $[2M+Na]^+$, (19)<br>162.08, $[M-H_2O+H]^+$, (46)	Maltose ($C_{12}H_{22}O_{11}$)	342.1162	365.11, $[M+Na]^+$, (100)<br>685.24, $[2M+H]^+$, (0.2)<br>707.22, $[2M+Na]^+$, (36)<br>729.21, $[2M+2Na-H]^+$, (0.1)
D-Glucosamine ($C_6H_{13}NO_5$)	179.0794	180.09, $[M+H]^+$, (100)<br>202.07, $[M+Na]^+$, (18)<br>359.17, $[2M+H]^+$, (49)<br>381.15, $[2M+Na]^+$, (23)<br>162.08, $[M-H_2O+H]^+$, (33)	Cellobiose ($C_{12}H_{22}O_{11}$)	342.1162	343.13, $[M+H]^+$, (0.3)<br>365.11, $[M+Na]^+$, (100)<br>685.24, $[2M+H]^+$, (2.2)<br>707.22, $[2M+Na]^+$, (38)
D-N-acetyl-galactosamine ($C_8H_{15}NO_6$)	221.0899	222.10, $[M+H]^+$, (0.01)<br>244.08, $[M+Na]^+$, (71)<br>266.06, $[M+2Na-H]^+$, (1)<br>465.17, $[2M+Na]^+$, (100)<br>487.15, $[2M+2Na-H]^+$, (1)	Xylose ($C_5H_{10}O_5$)	150.0528	173.04, $[M+Na]^+$, (100)<br>195.02, $[M+2Na-H]^+$, (2)<br>323.09, $[2M+Na]^+$, (81)<br>345.07, $[2M+2Na-H]^+$, (3)<br>367.06, $[2M+3Na-2H]^+$, (0.1)
D-N-acetyl-glucosamine ($C_8H_{15}NO_6$)	221.0899	222.10, $[M+H]^+$, (1)<br>244.08, $[M+Na]^+$, (68)<br>266.06, $[M+2Na-H]^+$, (2)<br>465.17, $[2M+Na]^+$, (100)<br>487.15, $[2M+2Na-H]^+$, (1)	Mannose ($C_6H_{12}O_6$)	180.0634	203.05, $[M+Na]^+$, (98)<br>225.03, $[M+2Na-H]^+$, (1.4)<br>383.11, $[2M+Na]^+$, (100)<br>405.10, $[2M+2Na-H]^+$, (4)<br>427.08, $[2M+3Na-2H]^+$, (0.2)
N-acetyl-neuraminic acid ($C_{11}H_{19}NO_9$)	309.1060	332.09, $[M+Na]^+$, (16)<br>354.08, $[M+2Na-H]^+$, (100)<br>376.06, $[M+3Na-2H]^+$, (0.5)<br>641.20, $[2M+Na]^+$, (0.4)<br>663.18, $[2M+2Na-H]^+$, (2)<br>685.16, $[2M+3Na-2H]^+$, (28)	L-Fucose ($C_6H_{12}O_5$)	164.0685	187.06, $[M+Na]^+$, (100)<br>209.04, $[M+2Na-H]^+$, (1)<br>351.13, $[2M+Na]^+$, (56)
D-Glucuronic acid ($C_6H_{10}O_7$)	194.0427	195.04, $[M+H]^+$, (2)<br>217.03, $[M+Na]^+$, (6)<br>239.01, $[M+2Na-H]^+$, (100)<br>433.06, $[2M+2Na-H]^+$, (1)<br>455.04, $[2M+3Na-2H]^+$, (50)	L-Iduronic Acid ($C_6H_{10}O_7$)	194.0427	217.03, $[M+Na]^+$, (100)<br>239.01, $[M+2Na-H]^+$, (42)<br>411.07, $[2M+Na]^+$, (7)
D0A4 ($C_{14}H_{20}NNaO_{14}S$)	481.0502	482.05, $[M+H]^+$, (1)<br>504.03, $[M+Na]^+$, (51)<br>526.02, $[M+2Na-H]^+$, (100)	D0A6 ($C_{14}H_{20}NNaO_{14}S$)	481.0502	482.06, $[M+H]^+$, (2)<br>504.04, $[M+Na]^+$, (45)<br>526.02, $[M+2Na-H]^+$, (100)
Ala-Ser-Ala ($C_9H_{16}N_3O_5$)	246.1090	247.14, $[M+H]^+$, (100)<br>269.12, $[M+Na]^+$, (29)<br>493.27, $[2M+H]^+$, (4)	Ala-Ser(O-α-GalNAc)-Ala ($C_{17}H_{31}N_5O_9$)	449.2122	450.22, $[M+H]^+$, (100)<br>472.20, $[M+Na]^+$, (14)<br>899.43, $[2M+H]^+$, (1)
ICA ($C_6H_9N_3OS$)	171.0466	172.05, $[M+H]^+$, (10)<br>194.04, $[M+Na]^+$, (100)<br>216.02, $[M+2Na-H]^+$, (2)<br>365.08, $[2M+Na]^+$, (33)			

1. The relative Intensity (%) value of observed ions are given in parentheses next to each complex ion. The most intense peak in a single stage MS spectrum is defined as 100.

Supplementary Table 3. Characteristic MS Peaks of 1:1 ICA-Carbohydrate complexes and their MS/MS products

(M denotes the corresponding carbohydrate molecule)

Analytes	Observed m/z	MS/MS Product Ion
ICA + carbohydrate	Mass of adduct ion (Intensity, S/N)	Mass of Product ion, (Intensity)
D-Galactose	374.10, [ICA+M+Na]$^+$, (41, 15417)	194.04, [ICA+Na]$^+$, (100)<br>203.05, [M+Na]$^+$, (87)
D-Glucose	374.10, [ICA+M+Na]$^+$, (0.9, 804)	194.04, [ICA+Na]$^+$, (100)<br>203.05, [M+Na]$^+$, (8)
	396.08, [ICA+M+2Na-H]$^+$, (0.2, 195)	216.02, [ICA+2Na-H]$^+$, (51)<br>225.03, [M+2Na-H]$^+$, (100)
D-Galactosamine	351.13, [ICA+M+H]$^+$, (55, 33497)	162.08, [M-H_2O+H]$^+$, (13)<br>172.05, [ICA+H]$^+$, (19)<br>180.09, [M+H]$^+$, (100)
	373.12, [ICA+M+Na]$^+$, (16, 10165)	194.04, [ICA+Na]$^+$, (46)<br>202.07, [M+Na]$^+$, (100)
D-Glucosamine	351.13, [ICA+M+H]$^+$, (43, 18890)	162.08, [M-H_2O+H]$^+$, (10)<br>172.05, [ICA+H]$^+$, (53)<br>180.09, [M+H]$^+$, (100)
D-N-acetyl-galactosamine	393.14, [ICA+M+H]$^+$, (0.3, 63)	172.05, [ICA+H]$^+$, (68)<br>222.10, [M+H]$^+$, (100)
	415.12, [ICA+M+Na]$^+$, (15, 3030)	194.03, [ICA+Na]$^+$, (0.2)<br>244.08, [M+Na]$^+$, (100)
	437.10, [ICA+M+2Na-H]$^+$, (0.5, 107)	244.08, [M+Na]$^+$, (47)<br>266.06, [M+2Na-H]$^+$, (100)
D-N-Acetyl-Glucosamine	415.13, [ICA+M+Na]$^+$, (53, 13720)	194.04, [ICA+Na]$^+$, (12)<br>244.08, [M+Na]$^+$, (100)
N-acetyl-neuraminic acid	503.12, [ICA+M+Na]$^+$, (0.03, 15)	194.03, [ICA+Na]$^+$, (42)<br>332.08, [M+Na]$^+$, (39)
	525.12, [ICA+M+2Na-H]$^+$, (3.1, 1569)	354.08, [M+2Na-H]$^+$, (100)
	547.10, [ICA+M+3Na-2H]$^+$, (0.03, 17)	332.09, [M+Na]$^+$, (8)<br>354.08, [M+2Na-H]$^+$, (100)
D-Glucuronic acid	366.08, [ICA+M+H]$^+$, (7, 2658)	194.04, [ICA+Na]$^+$, (100)<br>195.04, [M+H]$^+$, (73)
	388.08, [ICA+M+Na]$^+$, (0.1, 43)	194.04, [ICA+Na]$^+$, (100)
D0A4	675.09, [ICA+M+Na]$^+$, (0.01, 3)	194.01, [ICA+Na]$^+$, (53)<br>504.03, [M+Na]$^+$, (100)
	697.07, [ICA+M+2Na-H]$^+$, (0.1, 42)	194.01, [ICA+Na]$^+$, (14)<br>526.02, [M+2Na-H]$^+$, (100)
Ala-Ser-Ala	418.19, [ICA+M+H]$^+$, (1.3, 539)	172.06, [ICA+H]$^+$, (1)<br>247.14, [M+H]$^+$, (100)
	440.17, [ICA+M+Na]$^+$, (0.3, 152)	269.12, [M+Na]$^+$, (100)

Analytes	Observed m/z	MS/MS Product Ion
ICA + carbohydrate	Mass of adduct ion (Intensity, S/N)	Mass of Product ion, (Intensity)
D-α ^{M}Glu	388.12, [ICA+M+Na]$^+$, (2.1, 1494)	194.04, [ICA+Na]$^+$, (100)<br>217.07, [M+Na]$^+$, (15)
D-β ^{M}Glu	366.08, [ICA+M+H]$^+$, (6, 1935)	172.05, [ICA+H]$^+$, (3)<br>194.05, [ICA+Na]$^+$, (100)<br>195.04, [M+H]$^+$, (72)
	388.12, [ICA+M+Na]$^+$, (65, 21034)	194.04, [ICA+Na]$^+$, (100)<br>217.07, [M+Na]$^+$, (14)
Maltose	514.17, [ICA+M+H]$^+$, (1.5, 451)	172.06, [ICA+H]$^+$, (100)
	536.15, [ICA+M+Na]$^+$, (9.5, 3093)	194.04, [ICA+Na]$^+$, (2)<br>365.11, [M+Na]$^+$, (100)
Cellobiose	514.17, [ICA+M+H]$^+$, (1.2, 838)	172.06, [ICA+H]$^+$, (100)
	536.15, [ICA+M+Na]$^+$, (1.1, 846)	365.11, [M+Na]$^+$, (100)
Xylose	344.09, [ICA+M+Na]$^+$, (9.0, 5451)	173.04, [M+Na]$^+$, (10)<br>194.03, [ICA+Na]$^+$, (100)<br>195.04, [M+2Na-H]$^+$, (5)
	366.08, [ICA+M+2Na-H]$^+$, (2.6, 1553)	194.03, [ICA+Na]$^+$, (100)<br>195.04, [M+2Na-H]$^+$, (12)
	388.06, [ICA+M+3Na-2H]$^+$, (0.1, 65)	194.03, [ICA+Na]$^+$, (13)<br>216.02, [ICA+2Na-H]$^+$, (100)
Mannose	374.10, [ICA+M+Na]$^+$, (20, 10478)	194.03, [ICA+Na]$^+$, (78)<br>203.05, [M+Na]$^+$, (87)
	396.08, [ICA+M+2Na-H]$^+$, (0.5, 291)	216.02, [ICA+2Na-H]$^+$, (22)<br>225.03, [M+2Na-H]$^+$, (100)
L-Fucose	336.12, [ICA+M+H]$^+$, (0.1, 39)	172.06, [ICA+H]$^+$, (100)
	358.10, [ICA+M+Na]$^+$, (8, 4586)	187.06, [M+Na]$^+$, (4)<br>194.04, [ICA+Na]$^+$, (100)
L-Iduronic Acid	388.08, [ICA+M+Na]$^+$, (0.3, 211)	194.04, [ICA+Na]$^+$, (86)<br>217.03, [M+Na]$^+$, (100)
	410.06, [ICA+M+2Na-H]$^+$, (0.7, 489)	194.04, [ICA+Na]$^+$, (4)<br>217.03, [M+Na]$^+$, (7)<br>239.01, [M+2Na-H]$^+$, (100)
D0A6	675.09, [ICA+M+Na]$^+$, (0.01, 11)	194.04, [ICA+Na]$^+$, (14)<br>504.04, [M+Na]$^+$, (100)
	697.07, [ICA+M+2Na-H]$^+$, (0.02, 22)	194.04, [ICA+Na]$^+$, (16)<br>526.02, [M+2Na-H]$^+$, (100)
Ala-Ser(O-α-GalNAc)-Ala	621.27, [ICA+M+H]$^+$, (0.03, 13)	450.22, [M+H]$^+$, (100)
	643.24, [ICA+M+Na]$^+$, (0.01, 6)	472.20, [M+Na]$^+$, (100)

The Relative Intensity (I%) and Signal to Noise Ratio (S/N) values are given in parentheses respectively next to each complex ion in observed m/z column. I% values are reported in parentheses next to each complex ion in MS/MS product ion column. The most intense peak is considered as 100.

Supplementary Table 4. Characteristic MS Peaks of 2:1 ICA-Carbohydrate complexes and their MS/MS products (M denotes the corresponding carbohydrate molecule)

Analyte	Observed m/z	MS/MS Product Ion	Analyte	Observed m/z	MS/MS Product Ion
ICA + carbohydrate	Mass of adduct ion, (Intensity, S/N)	Mass of Product ion, (Intensity)	ICA + carbohydrate	Mass of adduct ion, (Intensity, S/N)	Mass of Product ion, (Intensity)
D-Galactose	545.13, [2ICA+M+Na]$^+$, (0.1, 35)	365.06, [2ICA+Na]$^+$, (87)	D-α ^{M}Glu	559.08, [2ICA+M+Na]$^+$, (0.02, 14)	Not measured
D-Glucose	Not measured		D-β ^{M}Glu	559.14, [2ICA+M+Na]$^+$, (0.04, 12)	365.06, [2ICA+Na]$^+$, (87)
D-Galactosamine	522.16, [2ICA+M+H]$^+$, (0.1, 60)	180.09, [M+H]$^+$, (5)	Maltose	Not measured	
D-Glucosamine	Not measured		Cellobiose	Not measured	
D-N-acetyl-galactosamine	586.15, [2ICA+M+Na]$^+$, (1.3, 264)	244.08, [M+Na]$^+$, (12)	Xylose	537.11, [2ICA+M+2Na-H]$^+$, (0.01, 4)	194.03, [ICA+Na]$^+$, (52)
	608.13, [2ICA+M+2Na-H]$^+$, (0.1, 11)	216.02, [ICA+2Na-H]$^+$, (100)<br>244.08, [M+Na]$^+$, (97)		559.09, [2ICA+M+3Na-2H]$^+$, (0.03, 19)	194.03, [ICA+Na]$^+$, (9)<br>216.02, [ICA+2Na-H]$^+$, (15)
D-*N*-acetyl-glucosamine	Not measured		Mannose	567.11, [2ICA+M+2Na-H]$^+$, (0.03, 18)	216.02, [ICA+2Na-H]$^+$, (100)
N-Acetyl-neuraminic acid	674.17, [2ICA+M+Na]$^+$, (0.05, 24)	332.08, [M+Na]$^+$, (100)<br>354.08, [M+2Na-H]$^+$, (50)	L-Fucose	529.13, [2ICA+M+Na]$^+$, (0.01, 13	187.06, [M+Na]$^+$, (34)<br>194.04, [ICA+Na]$^+$, (100)
	696.15, [2ICA+M+2Na-H]$^+$, (0.1, 53)	354.08, [M+2Na-H]$^+$, (100)			
D-Glucuronic acid	559.11, [2ICA+M+Na]$^+$, (0.02, 6)	194.03, [ICA+Na]$^+$, (15)	L-Iduronic Acid	581.11, [2ICA+M+2Na-H]$^+$, (0.1, 107)	239.01, [M+2Na-H]$^+$, (100)
D0A4	868.13, [2ICA+M+2Na-H]$^+$, (0.02, 23)	194.01, [ICA+Na]$^+$, (48)<br>526.02, [M+2Na-H]$^+$, (100)	D0A6	868.12, [2ICA+M+2Na-H]$^+$, (0.01, 13)	194.04, [ICA+Na]$^+$, (43)<br>526.02, [M+2Na-H]$^+$, (100)
Ala-Ser-Ala	611.20, [2ICA+M+Na]$^+$, (0.03, 22)	269.12, [M+Na]$^+$, (100)	Ala-Ser(O-α-GalNAc)-Ala	792.33, [2ICA+M+H]$^+$, (0.04, 24)	450.22, [M+H]$^+$, (100)
				814.31, [2ICA+M+Na]$^+$, (0.03, 18)	472.20, [M+Na]$^+$, (100)

The Relative Intensity (I%) and Signal to Noise Ratio (S/N) values are given in parentheses respectively next to each complex ion in observed m/z column. I% values are reported in parentheses next to each complex ion in MS/MS product ion column. The most intense peak is considered as 100

Characterization of functionalized palladium substrates

Thickness. We characterized the substrate with ellipsometry. The bare palladium substrate was annealed by hydrogen flame and its thickness was measured using Gaertner L 123b Ellipsometer (Gaerner Scientific Corporation) prior to functionalization. A refractive index of 1.50 was assumed for the organic thin film.[1] The monolayer measured 9.10±0.41 Å in thickness on average from five measurements in different locations of two samples. The ICA molecule was estimated by ChemDraw 3D to be ~8.3 Å long.

Contact angle. Static water contact angles were measured for hydrogen annealed palladium substrate prior to SAM formation and for functionalized substrate after SAM formation using an Easydrop Drop Shape Analysis System (KRÜSS GmbH, Hamburg). Volume of each water droplet for static contact angle measurements was 1 µL. 5-6 measurements were taken on different locations of each functionalized and bare palladium substrates. The contact angle for the bare palladium substrate was 8.3 ± 2.0°, for the ICA monolayer 33.1 ± 5.1°

FTIR spectra. The FTIR spectra were recorded using a Nicolet 6700 FT-IR (Thermo Electron Corporation) equipped with a MCT detector at a 4 cm^{-1} resolution in a range from 4000 to 650 cm^{-1}, with an attenuated total reflection accessory (Smart Orbit, Thermo Electron Corporation) for an ICA powder with 128 scans (3500-1000 cm^{-1} shown in Supplementary Figure 2, **a**) and with a surface grazing angle accessory (Smart SAGA, Thermo Electron Corporation) with 256 scans for the ICA monolayer [1] (3500-1000 cm^{-1} shown in Supplementary Figure 2, **b**). The ICA powder sample gave a broad band in the region of 3400-2800 cm^{-1}, indicating the intermolecular hydrogen-bonding interactions between the ICA molecules; in contrast, the ICA monolayer shows very sharp peaks in the same region due to the removal of the intermolecular hydrogen bonds. Both spectra show the vibrations of the amide function in the region of 1700-1600 cm^{-1}.

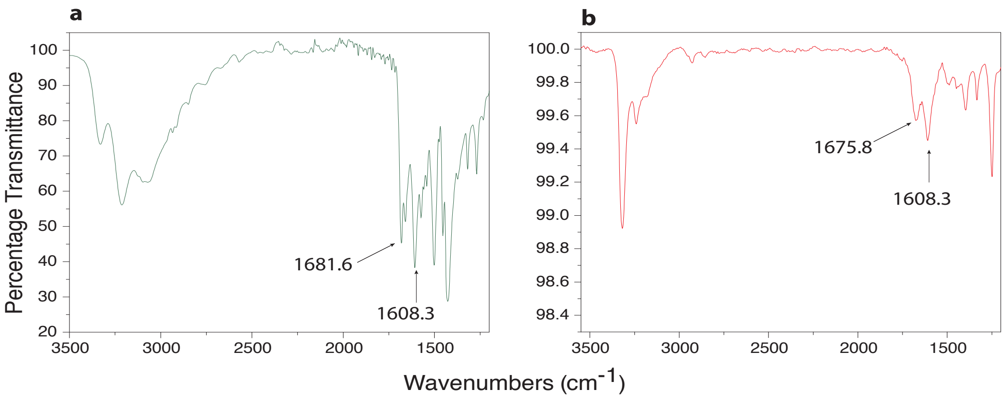

Supplementary Figure 2. FTIR spectra of (a) ICA powder and (b) ICA monolayer

X-ray photoelectron spectroscopy (XPS). XPS spectra were obtained using a VG ESCALAB 220i-XL photoelectron spectrometer and Al-Kα radiation (15keV) at 6 x 10E-10 mbar base pressure. The high resolution spectra of C(1s), Pd(3d), N(1s) and S(2p) were recorded at a pass energy of 20 eV and wide scan spectra were obtained at pass energy of

150 eV. The data analysis was carried out using CasaXPS software. C(1s), N(1s), and S(2p) core peaks were fitted and the ICA element ratio was calculated through area integral of peaks. Supplementary Table 5 shows the found elemental ratio, which is close to the calculated ratio.

Supplementary Table 5. Element compositions of the imidazole monolayer from XPS

Element	Atomic Percentage (%)	Found elemental ratio	Calculated elemental ratio
S 2p	5.17	1	1
C 1s	26.77	5.2	6
N 1s	12.22	2.4	3

STM experimental details and conditions

Determination of servo gain value

The effect of altering the gain values was determined by noise spectrum measurements (Power Spectral Density) under various gain values. Supplementary Figure 3 shows that servo control distorts the signal. With 0.1 for the integral and proportional gain, the spectral range under 30 Hz (corresponding 33 ms) was suppressed, resulting in distortion of only a few of the longer spikes. The tunneling current was Fourier transformed and plotted as a spectral density calculated by

$$PSD = \frac{2}{N \cdot \Delta t} \frac{Re^2 + Im^2}{f}$$

where $N = 50{,}000$ and $\Delta t = 20\ \mu s$.

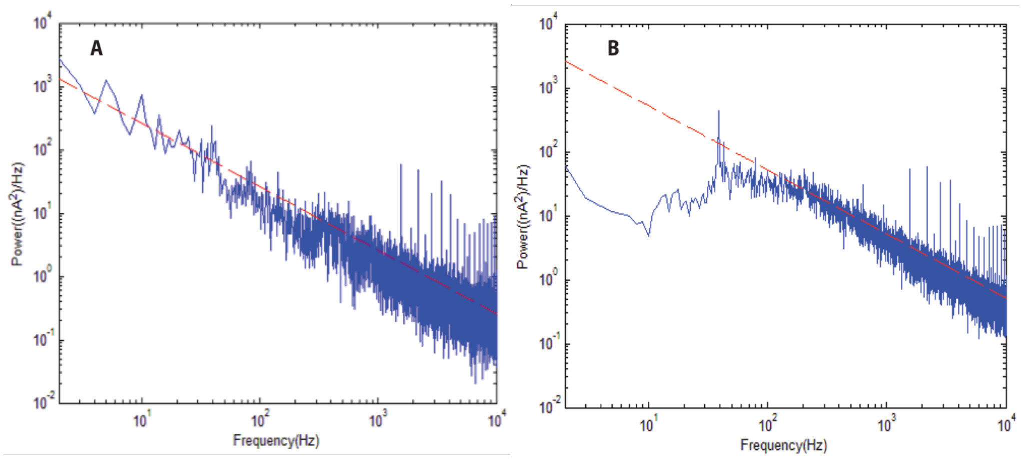

Supplementary Figure 3. Noise spectrum (a) without servo control and (b) with servo control. Blue lines are the noise spectrum, and red lines are fits to 1/f spectrum.

Supplementary Table 6. Frequency of current spikes with different electrodes in phosphate buffer, Galactose and Glucose solution

	Control	Galactose	Glucose
**Bare electrodes**	0	0.33	0.34
**ICA-electrodes**	0	3.03	1.22
Unit: peaks/second			

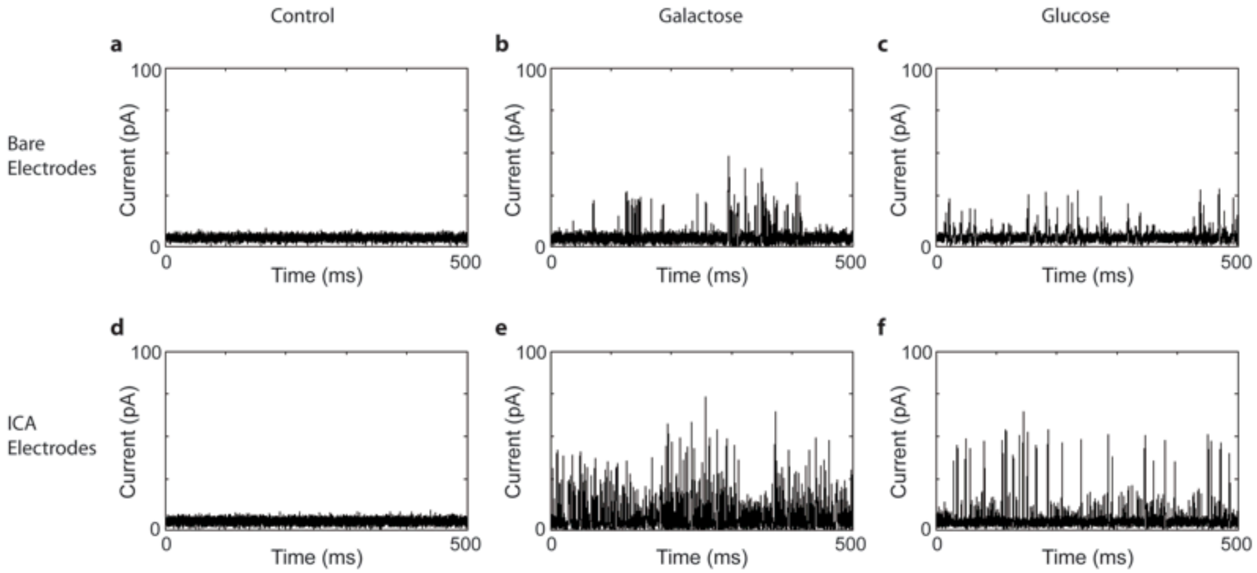

Supplementary Figure 4. RT Spectra measured with bare electrodes (a, b, c) and with ICA functionalized electrodes (d, e, f) in different solutions

RT spectra of carbohydrates

Supplementary Figure 5 shows exemplary RT spectra of all saccharides we have measured.

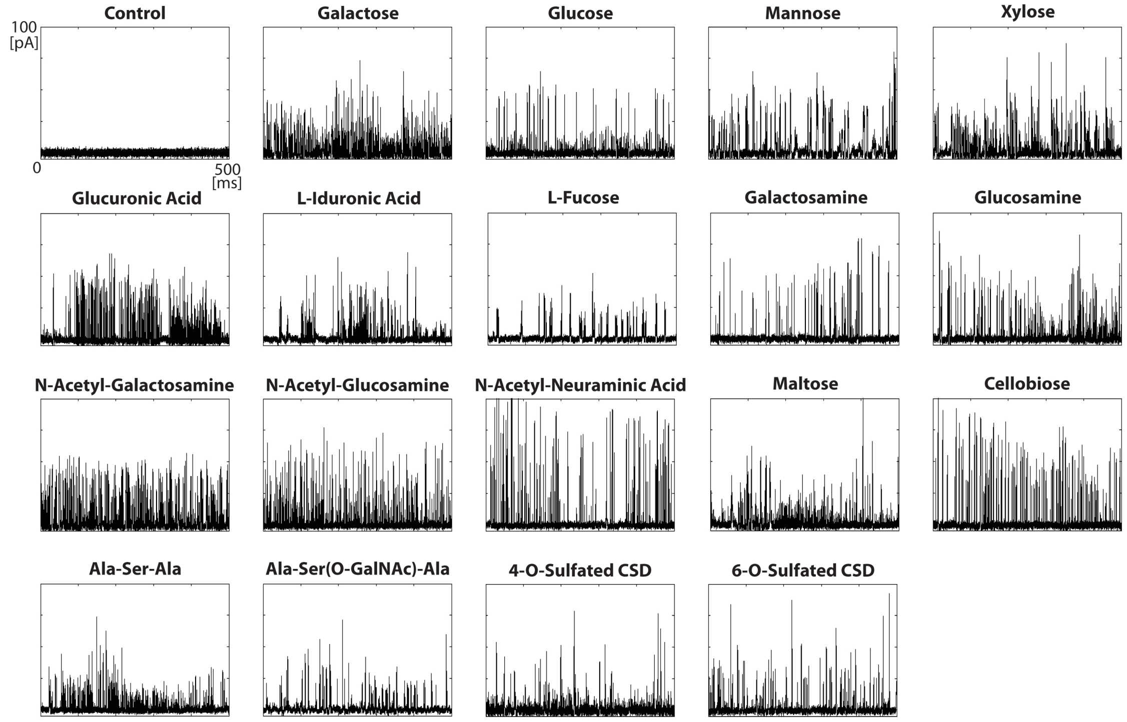

Supplementary Figure 5. Representative RT Spectra of saccharides measured with ICA functionalized electrodes

Data analysis

Feature Extraction

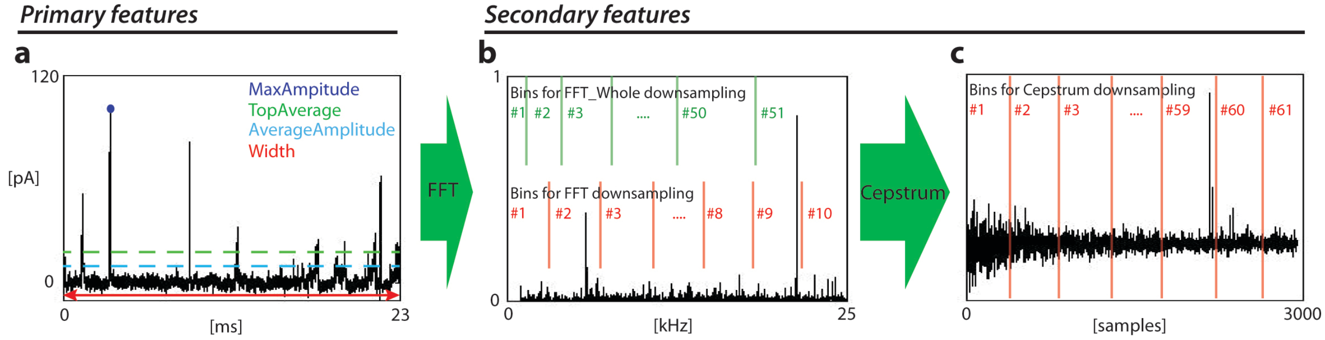

Supplementary Figure 6. Feature definitions for cluster features

(a) Primary features in time domain; (b) secondary features in frequency domain, (c) secondary features in cepstrum domain.

Feature descriptions

<u>*Classical features:*</u>

- Peak/Cluster Width is the time the molecule is producing current above the baseline.
- Peak/Cluster Roughness is the standard deviation of the cluster or peak.
- Peak/Cluster Average Amplitude is the mean current of the peak/cluster.
- Peak/Cluster Top Average is the mean of the thresholded currents, i.e. only those values above the threshold are used to calculate the average.
- Peak frequency describes the peaks per second.
- Peaks In Cluster is the number of signal peaks in the cluster.

<u>*Noise:*</u>

- Peak/Cluster FFT is the normalized power spectrum, downsampled into 10/61 equal frequency bands.
- Peak/Cluster FFT Whole is the normalized power spectrum, denoised with Weiner filtering, and then downsampled into 51 frequency bands in which the bin sizes are spaced according to a power 2 square law, as the greater amount of information is contained in the lower frequency bands.
- Peak/Cluster iFFT is the sum of three frequency power spectrum components; Low is the first three bins, Medium is three bins in middle, and High is the last three bins of the spectrum.
- Peak/Cluster Total Power is the sum of the power spectrum.
- Cluster Frequency Maximum (n) is the nth dominate frequency on the power spectrum after correction for 1/f noise.

<u>*Peak shape proxy:*</u>

- Cluster Cepstrums are created by taking a power spectrum, correcting for 1/f noise and then performing another power spectrum. The spectrum is downsampled into 61 bins. These

are typically used to determine harmonics in a signal trace, but here serve as a proxy for the shape of the peaks.
- Cluster High/low features are created by dividing the sum of the top quarter of the power spectrum by the sum of the bottom quarter of the spectrum. This serves as a nice proxy for the sharpness of the peaks.
- The Odd/Even Ratio is the ratio of the sum of the odd power spectrum components vs the even power spectrum components.

Supplementary Table 7. Principle component vectors used in Figure 1j

Coefficients of Dim 4	Coefficients of Dim 8	Components
0.033195	0.101947	'C_clusterCepstrum1'
-0.0618	0.524793	'C_clusterFFT26'
-0.09298	-0.24804	'C_clusterFFT28'
-0.03694	0.130899	'C_clusterFFT_Whole29'
-0.00759	-0.01849	'C_clusterFFT55'
0.85614	0.807382	'C_clusterCepstrum53'
0.02719	0.203706	'C_totalPower'
-0.00923	-0.02486	'P_Even_FFT'
-0.07694	-0.11248	'C_clusterFFT2'
-0.08426	-0.13207	'C_clusterCepstrum10'
0.07275	0.088757	'C_clusterCepstrum31'
-0.14972	-0.68808	'C_clusterFFT_Whole28'
-0.07168	0.214511	'P_peakFFT3'
0.024927	0.134196	'C_averageAmplitude'
-0.00793	-0.02122	'C_clusterFFT48'
0.007242	0.011516	'C_clusterFFT25'
0.008747	0.09376	'C_clusterFFT_Whole44'
0.046045	0.20501	'C_clusterCepstrum57'
-0.00739	-0.01951	'C_clusterFFT39'
0.081674	-0.27008	'C_clusterCepstrum24'
-0.0083	-0.02218	'C_clusterCepstrum5'
0.43855	0.465232	'P_averageAmplitude'
-0.00736	-0.01837	'C_clusterCepstrum21'
0.038176	0.128548	'C_clusterFFT20'
-0.00825	-0.02142	'C_clusterFFT10'
-0.00756	-0.02137	'C_clusterFFT_Whole37'
-0.00802	-0.02264	'C_clusterCepstrum13'
-0.01355	0.065165	'C_clusterCepstrum47'
-0.00776	-0.02194	'C_freq_Maximum_Peaks4'

Effects of ICA on anomer distribution of glucose in the equilibrium and its mutarotation rate.

We measured anomeric distribution of ^{13}C-labeled glucose (0.1 mM) in 50 mM phosphate, pH 7.4 and 150 mM NaCl (PBS) using ^{1}H-^{13}C heteronuclear single quantum coherence

spectroscopy (HSQC). The experiment was carried out on a Bruker 600 MHz NMR spectrometer equipped with a Prodigy probe. The effect of ICA on the equilibrium anomer distribution was analyzed determining the α/β ratios in the presence and absence of 1 mM ICA. The rate of mutarotation was measured by observing the conversion of α-D-glucose to β-D-glucose immediately after the dissolution. Specifically, ^{13}C-labeled α-D-glucose was dissolved in PBS containing either 1 mM ICA or no ICA. The change in distribution of α/β anomers was measured by acquiring 1D ^{1}H-^{13}C HSQC experiment every two minutes for sixty minutes. The rates of conversion are directly proportional to the rates of intensity decrease in the ^{1}H-^{13}C peaks for the α anomer (Supplementary Figure 7, **a**). The ratios of the glucose anomers were determined by measuring the volumes of H1-C1 peaks for α and β anomers (Supplementary Figure 7, **b** and **c**). The sample was allowed to equilibrate for 24 hours before data acquisition.

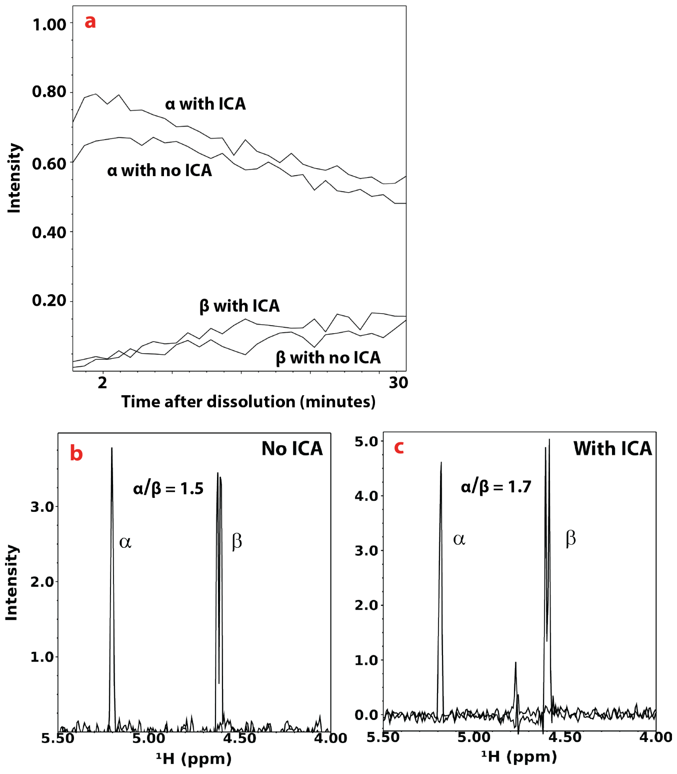

Supplementary Figure 7. Effects of ICA on mutarotation rates and anomeric ratios of glucose
(**a**) The mutarotation rates of α/β anomers of glucose in the absence and presence of ICA; (**b**) the α/β anomer ratio of glucose in the absence of ICA, and (**c**) the α/β ratio of glucose in the presence of ICA

RT signal frequency from α-^{M}Glu as a function of concentration

The α-^{M}Glu was dissolved in the phosphate buffer to make a 1 mM stock solution, which was diluted to various concentrations from 500 μM to 100 pM. For each measurement, an analyte solution (200 μL) was injected into the liquid cell using a syringe attached to a micro filter. After the measurement, the liquid cell and electrodes were rinsed with the phosphate buffer solution (3 mL) through the fluidic channels to obtain a clean control signal. A pair of electrodes was able to carry out three measurements with different concentrations from lower to higher concentration and the measurement at each concentration was repeated at least 3 times (Supplementary Table 8). The isotherm absorption data were analyzed in software OriginPro 2016 using the Levenberg-Marquardt algorithm for fitting to a Langmuir equation:
$\mathbf{f(x)} = \mathbf{a} * (\mathbf{b} \cdot \mathbf{x}) / (1 + \mathbf{b} \cdot \mathbf{x})$

Supplementary Table 8. RT signals frequency of α-^{M}Glu at different concentrations

	Average peak counts (peaks/sec)								
Conc.	Runs					Average	STD-mean	Normalized	STD-mean
100 pM	0.02	0.08	0.05			0.050	0.017	0.000	0.008
1 nM	0.04	0.13	0.17			0.113	0.038	0.030	0.018
10 nM	0.12	0.1	0.13	0.16	0.22	0.146	0.021	0.046	0.010
100 nM	0.33	0.35	0.57	0.41		0.415	0.054	0.174	0.026
1 μM	0.86	1.17	0.88			0.970	0.100	0.437	0.048
10 μM	1.89	1.61	1.78			1.760	0.081	0.813	0.039
100 μM	2.33	1.99	1.99	1.75		2.015	0.119	0.934	0.057
250 μM	2.35	2.08	2.2	1.7		2.083	0.139	0.966	0.066
500 μM	2.26	1.8	2.4			2.153	0.181	1.000	0.086

Supplementary Table 9. RT signal frequency of α-^{M}Glu, β-^{M}Glu, Galactose, and Glucose at 100 μM

	α-^{M}Glu	β-^{M}Glu	Galactose	Glucose
Run #1	1.34	1.27	3.25	2.57
Run #2	2.40	1.15	5.14	1.39
Run #3	1.67	0.86	8.01	1.05
Run #4	3.07	1.36	-	-
Average	2.12	1.16	5.47	1.67

Surface Plasmon Resonace (SPR) experiments

A gold chip was immersed into an absolute ethanol solution of ICA (100 μM) for 24 h, followed by rinsing with absolute ethanol and drying with a nitrogen flow, and used immediately. The instrument Bi 2000 from Biosensing Instrument was used for SPR

measurements. An ICA modified gold chip was mounted on the instrument and calibrated with 1% ethanol in a PBS buffer, pH 7.4. A solution of α-^{M}Glu (500 μM) was flowed onto the chip at a rate of 50 μl/min over a period of 1.5 min, followed by flowing the PBS buffer (Supplementary Figure 8). Association (k_{on}) and dissociation rate constants (k_{off}) were determined using built-in Biosensing Instrument SPR data analysis software version 2.4.6 (Supplementary Table 10).

Supplementary Table 10. Kinetic parameters of α-^{M}Glu on the ICA monolayer[1]

α -^{M}Glu	k_{on} ($M^{-1} s^{-1}$)	k_{off} (s^{-1})	K_d[2] (mM)	Res.sd
500 uM	23.05±1.34	0.09±0.01	4.00±0.14	10.2

1. Each datum listed is an average of two measurements.
2. $K_d = k_{off}/k_{on}$

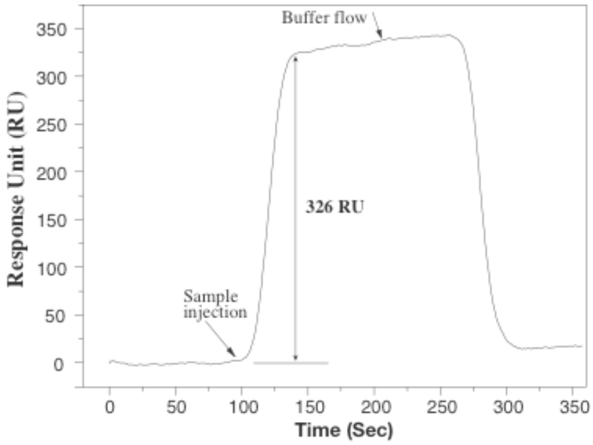

Supplementary Figure 8. SPR sensorgram of adsorption and desorption of α-^{M}Glu on the ICA monolayer

Supplementary Reference

1 Ulman, A. *An Introduction to Ultrathin Organic Films: From Langmuir--Blodgett to Self--Assembly*. (Academic Press, 1991).
